# Interest in and Willingness to Use PrEP: A Cross-Sectional Study of Individuals with Problematic Substance Use Residing in a High HIV Prevalence Jurisdiction

**DOI:** 10.1007/s10508-024-02936-z

**Published:** 2024-07-17

**Authors:** Angela M. Heads, Constanza de Dios, Kaixuan An, Jin H. Yoon, Robert Suchting, Adrienne Gilmore-Thomas, Joy M. Schmitz

**Affiliations:** Faillace Department of Psychiatry and Behavioral Sciences, McGovern Medical School, University of Texas Health Science Center at Houston, 1941 East Rd., BBSB Suite 1316, Houston, TX 77054 USA

**Keywords:** HIV prevention, PrEP, Substance use, Stigma, Southern US, DSM-5

## Abstract

Although it is an effective HIV prevention method, pre-exposure prophylaxis (PrEP) is underutilized in the Southern US. Many people who use drugs (PWUD) have increased susceptibility to HIV which could be lessened by using PrEP. Potential barriers to PrEP use include lack of awareness of PrEP, low knowledge about HIV prevention, low self-efficacy for HIV prevention, inaccurate risk perceptions, and anticipated stigma. The current study examined predisposing, enabling, and reinforcing factors that may predict interest in PrEP. The purpose of the current study was to explore factors associated with interest in and willingness to use daily oral and long acting injectable PrEP among sexually active adult PWUD. The data were collected from adult participants (*n* = 270) residing in Harris County, TX, who self-reported problematic substance use and who reported oral, anal, or vaginal sex in the six months prior to completing the survey. The survey was distributed and completed online via Qualtrics Panels in March of 2022 and included measures of PrEP and HIV knowledge, PrEP stigma, sexual health self-efficacy, experiences of discrimination, health literacy, and medical mistrust. The majority of participants reported circumstances or behaviors that increased their susceptibility to HIV. Findings indicated that PrEP user stereotypes and PrEP anticipated disapproval by others were associated with interest in using daily oral PrEP and willingness to use long acting injectable PrEP. These results provide insight into reasons for low PrEP uptake among PWUD who live in a high HIV prevalence jurisdiction. Implications for HIV prevention intervention are discussed.

**Supplementary Information:**

The online version contains supplementary material available at 10.1007/s10508-024-02936-z.

## Introduction

In 2019, the United States launched the Ending the HIV Epidemic (EHE) plan with the central goal of reducing new human immunodeficiency virus (HIV) cases by 90% by 2030 (Fauci et al., 2019). Included in this plan is the prioritization of certain sociodemographic groups and geographic areas noted to be disproportionately impacted by HIV. Although new HIV cases in the US have declined since the implementation of the EHE plan, Southern states continue to disproportionately account for approximately 51% of new HIV diagnoses each year in the US despite only 38% of the US population residing in those areas (Centers for Disease Control & Prevention, 2022).

A clinical target to address that may help further decrease new HIV diagnoses is substance use, which increases susceptibility to HIV infection, and interferes with engagement in HIV prevention efforts. Substance use is associated with direct (e.g., injection drug use and shared needles or injection equipment with a person living with HIV) and indirect (e.g., inhibition and compromised decision making resulting in behaviors such as inconsistent condom use and multiple sex partners of unknown status) HIV transmission modes (Shiau et al., 2017). Additionally, substance use (e.g., hazardous alcohol use and use of drugs such as cannabis, methamphetamine, opioids, and cocaine) can increase impulsivity, reduce inhibitions, impair judgment, increase susceptibility to sexual coercion, and reduce the ability to refuse unsafe sexual situations (Frohmader et al., 2010; Maguiña et al., 2013; Raj et al., 2007; Wang & Maher, 2019). Given the multiple factors contributing to increased susceptibility to HIV among people who use drugs (PWUD), it is important to gain a greater understanding of how these factors and others may impact decisions to use effective HIV prevention tools such as regular HIV testing and pre-exposure prophylaxis (PrEP).

PrEP was found to be safe and effective in populations of men who have sex with men (MSM) and people who inject drugs (PWID) (Murchu et al., 2022). Among heterosexual individuals, PrEP has an estimated efficacy of 64–84% (Murnane et al., 2013). Despite PrEP’s proven efficacy in reducing the likelihood of HIV infection in those exposed to the virus, PrEP remains underutilized among populations who stand to benefit the most. According to the CDC, only 30% of the 1.2 million people who could benefit from PrEP were prescribed it in 2021. As noted above, the South has the highest rate of new HIV cases, but unfortunately also has the lowest rates of PrEP coverage—PrEP users relative to the number of newly diagnosed individuals with HIV (Sullivan et al., 2019).

The barriers and facilitators influencing PrEP engagement among PWUD are not completely understood. Various models have been developed to identify factors associated with health behavior, including behaviors that increase susceptibility to HIV and engagement in health-promoting behaviors that can decrease the chances of contracting HIV. One well-known model is the PRECEDE model (Predisposing, Reinforcing, and Enabling Constructs in Educational/Environmental Diagnosis and Evaluation), which posits that health behaviors are influenced by three categories: predisposing factors, enabling factors, and reinforcing factors (Glanz et al., 2008). Predisposing factors are characteristics inherent to the individual or environment (e.g., knowledge, health literacy). Enabling factors include skills necessary to facilitate health behaviors which may include self-efficacy, or confidence in one’s ability to practice HIV prevention behaviors (Bandura, 1990; Bandura & Watts, 1996). Reinforcing factors are the rewarding or punishing outcomes (e.g., discrimination, stigma) resulting from various behaviors, such as PrEP use (Glanz et al., 2008). Specific to HIV prevention, knowledge about HIV and HIV prevention (predisposing factors), self-efficacy for HIV preventive behaviors (enabling factors), and HIV-related stigma (reinforcing factors) are important to improving our understanding of barriers and facilitators to PrEP initiation and adherence.

HIV prevention knowledge is often lacking among PWUDs. For example, injection drug use represents the third most common route of HIV transmission (CDC, 2022). However, nearly a third of people who use injection drugs (PWID) have not heard of PrEP (Jo et al., 2020; Sherman et al., 2019). For people who use drugs through non-injection routes, awareness is also generally low across the PrEP care continuum (Gebru et al., 2022; Torres et al., 2019). Low PrEP knowledge and awareness are important to address among PWUD, as concerns related to PrEP side effects, interactions with misused substances, and competing health priorities due to substance use represent barriers to PrEP interest and initiation (Biello et al., 2018). Low levels of PrEP awareness and knowledge indicate that current PrEP implementation strategies have yet to reach this vulnerable population.

HIV prevention self-efficacy increases engagement in health-promoting behaviors and is positively associated with HIV prevention knowledge, condom use, partner communication, and PrEP and antiretroviral treatment adherence (Brasileiro et al., 2023; Carter et al., 2022; Cianelli et al., 2017; Kang et al., 2004; Starks et al., 2022). Self-efficacy may be a modifiable skill that increases protective behaviors including PrEP initiation and adherence (Bazzi et al., 2019; Farmanfarmaee et al., 2018; Golub et al., 2019).

Stigma, both held by the individual or perceived to be held by others, also negatively impacts HIV prevention efforts. Individuals who themselves hold negative attitudes toward people living with HIV are less likely to engage in HIV prevention strategies such as HIV testing and PrEP use (Kalichman & Simbayi, 2003; Reif et al., 2017). Additionally, perceived stigma (beliefs that stigmatizing attitudes are held by others) has been observed to be a barrier to HIV testing (Babalola, 2007; Sullivan et al., 2020). A growing body of research supports stigma-reducing interventions and normalization of HIV testing as a means of increasing HIV testing uptake (Conserve et al., 2018; Yamanis et al., 2017).

In addition to its negative impact on regular HIV testing, perceived stigma is also a major barrier to PrEP interest, uptake, initiation, and continuation (Calabrese, 2020). Concerns regarding presumed sexual promiscuity, fear of being assumed to be living with HIV, the possibility of being misidentified as gay or involved in transactional sex, and worries about having a partner’s HIV status disclosed are among the stigmatizing aspects related to low PrEP use (Heads et al., 2021; Patel et al., 2016). For example, Chittamuru et al. (2020) found that increased stigma was associated with lower PrEP initiation intention in a sample of women in New York City and Philadelphia. The complex dynamics of stigma and trying to address PrEP use in target populations are illustrated by PrEP public health campaigns. Current PrEP public health campaigns that target stigmatized groups with disproportionately high HIV rates have yielded limited success, and in turn, may unfortunately perpetuate stigma and negatively affect groups that are already marginalized (Keene et al., 2021; Phillips II et al., 2020).

The purpose of the current study was to explore potential barriers to daily oral PrEP and long acting injectable (LAI) PrEP use among sexually active adults who self-report a diagnosis of substance use disorder or problematic substance use. We hypothesized that factors related to HIV and PrEP-related knowledge, self-efficacy, and stigma would emerge as significant predictors of interest in and willingness to use PrEP, thereby informing targeted preventive strategies for this population.

## Method

### Participants and Procedure

The data for this study were collected from 270 adult participants residing in the Harris County, TX area via an online, self-administered survey distributed by Qualtrics Panels in March of 2022. Participants who have agreed to participate in survey research by opting in to membership to Qualtrics Panels who meet initial criteria set by the researchers were invited to participate in a study of HIV prevention and substance use. Qualtrics Panels are a division of Qualtrics, a research software company which specializes in web-based survey data collection. Qualtrics Panels manages the deployment of the survey from beginning to end, including inviting eligible members of the panel to participate, monitoring quality of the survey (i.e., random or inconsistent responding, overuse of item non-response, and “speeding”—an indicator of responding without reading the questions and choices). Potential participants were targeted based on the following eligibility criteria: (1) ≥ 18 years of age; (2) have problematic substance use or diagnosed with a substance use disorder per DSM-5 criteria; (3) sexually active (anal or vaginal sex) within the past 6 months; (4) fluent in English. Potential participants who met eligibility were invited to participate in a study on substance use, sexual behaviors, knowledge about PrEP, and perceptions regarding PrEP use. Those who consented to participate were linked to the online survey. All study procedures were approved by the institutional review board of the research institution.

### Measures

*Background Characteristics* Participants provided basic demographic information including age, race/ethnicity, and sexual orientation. Additionally, participants were asked to answer “yes” or “no” regarding whether they had heard of PrEP prior to the current study. They also responded to two items regarding concerns about HIV: (1) “How worried are you about getting HIV or AIDS?” and “How worried are you that you may have already been exposed to the HIV or AIDS virus?” Participants indicated their level of worry on a Likert scale ranging from 0 (not at all) to 5 (extremely).

*Substance Use* To confirm eligibility for the study based on problematic substance use or a substance use disorder, participants were asked (1) “Have you used any alcohol or drugs (other than prescribed medications) within the past 6 months?” (2) “Have you ever been diagnosed with a substance use disorder?”; and (3)” Do you believe your alcohol and/or drug use have caused problems for you?” Additionally, participants completed the AUDIT-C (Bush et al., 1998) to assess levels of hazardous alcohol use and the DUDIT to assess problems related to other substance use.

*Willingness and Interest in PrEP* The primary outcomes were interest in taking daily oral PrEP (“I am interested in taking PrEP”) and willingness to take long acting injectable (LAI) PrEP (“I am willing to take LAI”). Each item was assessed using a 5-point Likert scale indicating degree of agreement (1 = Strongly Disagree, 2 = Disagree, 3 = Unsure, 4 = Agree, 5 = Strongly Agree).

*Stigma Regarding PrEP Use* The 8-item PrEP Anticipated Stigma Scale (PASS) measures anticipated social stigma about PrEP use. The scale generates two subscale scores: (1) PrEP User Stereotypes and (2) PrEP Disapproval by Others (Calabrese et al., 2018). After viewing a brief description of PrEP. respondents were asked the degree to which they agree or disagree with statements such as “People would assume I slept around if they knew I took PrEP” on a 4-point Likert scale (1-strongly disagree to 4-strongly agree). Higher scores on the PrEP User Stereotypes subscale represent stronger perceptions regarding stereotypes associated with people who use PrEP. Higher scores on the PrEP Disapproval by Others subscale represent higher anticipated disapproval from others should they learn of PrEP being used. The subscales demonstrated acceptable internal consistency (Cronbach’s alpha for PrEP User Stereotypes = 0.79 and for PrEP disapproval by others = 0.77).

*Experiences of Discrimination* Self-reported experiences of discrimination were measured using the Everyday Discrimination Scale (Forman et al., 1997). This scale assesses the frequency of discrimination in the respondent’s daily life. Participants were asked how often they experience each of nine items (e.g., “You are treated with less respect than other people are”) with response choices consisting of “almost every day,” “at least once a week,” “a few times a month,” “a few times a year,” less than once a year,” and “never.” Respondents choosing “a few times a year” or more often were asked a follow-up question “What do you think is the main reason for these experiences?” with answer choices including race, gender, age. The score range for the Everyday Discrimination Scale is 9–54 with higher scores indicating more frequent discrimination experiences. Cronbach’s alpha for the scale in the current study was 0.91.

*Self-Efficacy for HIV Prevention* The Sexual Self-Efficacy Questionnaire developed by Smith et al. (1996) is a 9-item measure assessing a participant’s comfort with specific HIV and sexually transmitted infection protective behaviors (e.g., “Please indicate how sure or unsure you are about your ability to refuse to have sex with someone you didn't know very well”). The items were measured on a 5-point Likert scale (1 = not at all sure, 5 = very sure) with higher scores representing greater self-efficacy. Internal consistency was acceptable (Cronbach’s alpha = 0.89).

*Medical Mistrust* Medical mistrust was assessed using the Medical Mistrust Index (LaVeist et al., 2009), a 7-item scale created to measure mistrust in healthcare organizations. Participants indicate the degree to which they agree with statements such as “Patients have sometimes been deceived or misled by health care organizations,” on a 5-point Likert scale ranging from 1 (strongly disagree) to 5 (strongly agree), yielding a possible score between 7 and 35 with higher scores indicating greater medical mistrust. Cronbach’s alpha for the scale in the current study was 0.83.

*Health Literacy* Participants completed the 10-item HIV Knowledge Questionnaire (Carey & Schroder, 2002) consisting of true/false statements regarding knowledge of HIV transmission and risk. For each item, a score of 1 was assigned for correct answers and 0 for incorrect answers or indications that the respondent did not know the answer. Correct answers were summed to generate an overall score for each participant with higher scores indicating greater HIV knowledge. Additionally, participants completed a 6-item measure of PrEP knowledge consisting of statements in which the respondent was asked to indicate whether the items were true or false with higher scores indicating greater knowledge about PrEP (Heads, 2020). For example, participants are asked whether the statement “A person who suspects they have been exposed to HIV can take PrEP once to prevent HIV from taking hold and spreading through the body” is true or false or to indicate that they do not know. To assess general health literacy, the Calgary Charter on Health Literacy Scale (Pleasant et al., 2018) consists of five items. The scale instructs respondents “Please tell me on the following scale from Never to Always how often you engage in the following tasks?” followed by health information related tasks such as “Find or look for health information.” The scores range from 5 to 20. Internal consistency for the current study was acceptable (Cronbach’s alpha = 0.81).

### Data Analytic Plan

Analyses were performed in R (R Core Team, 2023). GLM (generalized linear model) regression tested each of the outcomes, interest in PrEP, and willingness to take LAI, as a function of each of the eight primary predictors in separate models (simple regressions). The eight primary predictors were: PrEP user stereotypes, expected PrEP disapproval by others, experiences of discrimination, medical mistrust, knowledge of HIV risk, knowledge of PrEP, health literacy, and self-efficacy.

The outcomes were then fit as a function of all eight predictors in multiple regression as follows. Primary predictors of interest were first classified according to the PRECEDE categories of predisposing factors: Knowledge of HIV risk, Knowledge of PrEP, and Health literacy; enabling factors: Self-Efficacy; and reinforcing factors: Discrimination and stigma (PrEP user stereotypes, PrEP disapproval from others, Experiences of discrimination, and Medical mistrust. The aim of this method was to identify best-fitting predictors from each category (Tier one) and to characterize the effect of the most impactful predictors identified in Tier one on the outcomes (Tier two). Stepwise regression (specifying both forward and backward directions via the stepAIC() function of the MASS package) (Venables, 2002) was performed in each predictor category separately to determine the most impactful predictors (Tier one). Tier one “winning variables” were determined using model fit rather than statistical significance. The stepwise approach compared the Akaike information criterion (AIC) improvements from dropping or adding each candidate variable that leads to the smallest AIC (Venables, 2002) until no further improvements can be made. Self-efficacy was always retained in this step as it was the sole variable in its own group. Tier one analyses were repeated in models adjusting for potentially confounding variables (described below). In all cases, decisions about retained predictors were obtained from the adjusted models and are shown here. (Unadjusted models are in Tables S2 and S3 of the Supplementary Materials.)

The retained predictors from each category in Tier one were then entered into a full regression to test their contributions to the model effect on outcomes in the presence of others (Tier two). Tier two analyses were performed again in models adjusting for relevant confounding variables. Most impactful predictors in Tier two were determined using statistical significance (*p* < 0.05).

All models covaried PrEP awareness when predicting interest in PrEP; LAI awareness was covaried when predicting willingness to take LAI. Additionally, demographic, substance use, and HIV prevention characteristics (age, sex, sexual orientation, Hispanic ethnicity, race, education, insurance, DUDIT score, worry about HIV, condom use, HIV ever tested, HIV last tested) were assessed as potential confounders using established procedures (Assmann et al., 2000; Pocock et al., 2002). A characteristic was considered a confounder if it covaried significantly with both the predictor of interest and the outcome. When testing as a confounder for multiple regressions that included a subset of the eight primary predictors, a characteristic was deemed a confounder if it covaried with at least one of the predictors of interest in the current model and the outcome. Characteristics deemed confounders were included as covariates in adjusted models (see Tables S1 of Supplementary Materials for summary of confounder relationships with primary outcomes and predictors). For simple regressions, unadjusted (no covariates with the exception of PrEP interest or LAI awareness) and adjusted models (with added covariates) were compared as an assessment of sensitivity. If relationships between primary predictors and outcomes in the adjusted models did not change in statistical significance from the unadjusted models, only the unadjusted models were reported. Otherwise, both models were reported and the confusion was described. For tier one multiple regressions, the adjusted models were used to determine winning variables. For tier two analyses, both unadjusted and adjusted models were compared.

## Results

Participant characteristics (*n* = 270) are summarized in Table 1. Most participants identified as White (60%), 25.9% identified as African American or Black, and 31.1% identified as Hispanic/Latinx/a/o. The mean age of participants was 34.0 years (SD = 9.4) and the majority identified as women (55.6%). All participants reported alcohol or other drug use within the six months prior to completing the survey. Nearly one-third of participants (31.5%) reported that they have been diagnosed with a substance use disorder, 65.2% reported that they believed their use of alcohol and or drugs have caused problems for them, and 42.6% reported that they believed that they needed treatment for their substance use. According to scores on the AUDIT-C, 67.8% scored above the cutoff for moderate to high alcohol risk. Based on DUDIT scores, 93.3% were above the cutoff for probable drug-related problems or probable substance use disorder.

**Table 1:** Sample characteristics (n = 270)

Demographic	Mean (SD)	*N*	%
*Age* (in years)	34.0 (9.37)		
Gender identity			
Woman		150	55.6
Man		114	42.2
Genderqueer/gender non-conforming		5	1.9
Other		1	0.4
*Ethnicity*			
Hispanic/Latinx		84	31.1
*Race*			
Black/African American		70	25.9
White		162	60.0
More than one race		13	4.8
Asian		8	3.0
American Indian/Native American		4	1.5
Pacific Islander		2	0.7
Other		11	4.1
*Sexual Orientation*			
Straight		206	76.3
Bisexual		34	12.6
Gay		8	3.0
Queer		2	0.7
Questioning		5	1.9
Other		7	2.6
*Condom Use*			
All of the time		49	18.1
Most of the time		47	17.4
Some of the time		59	21.9
None of the time		115	42.6
*Previous HIV Test*			
Never		94	34.8
*Ever Heard of PrEP*			
Yes		129	47.8
*Aware of LAI PrEP*			
Yes		58	21.5
*Sex Risk (past 6 months)*			
Sex while intoxicated or high		124	45.9
Condomless anonymous sex		47	17.4
Sex in exchange for drugs		79	29.3
Sex selling		50	20.4
Sex purchasing		48	17.8
*Reported Substances*			
Cannabis		184	68.1
Cocaine		60	22.2
Methamphetamine		60	22.2
Sedatives		56	20.7
Prescription Stimulants		44	16.3
Hallucinogens		40	14.8
Prescription Opioids		40	14.8
Street Opioids		20	7.4
Other		17	3.7
Inhalants		10	3.7

Participants reported sexual behaviors that are indicators for PrEP use according to the CDC guidelines including unprotected (i.e., condomless) anonymous sex and sex with a person of unknown HIV status (see Table 2).

**Table 2:** PrEP indicators

	Never (%)	More than 12 months ago (%)	Past 12 months (%)	Past 6 months (%)
I had unprotected anonymous sex	40.0	30.0	12.6	17.4
I had vaginal or anal sex while intoxicated and/or high on drugs	25.9	18.9	9.3	45.9
I had vaginal or anal sex with a person whose HIV status I did not know	59.6	18.9	7.8	13.7
I had vaginal or anal sex with a man who has sex with other men	73.3	10.0	4.8	11.9

About half of participants reported that they had never heard of PrEP (52.2%), 32.7% of participants reported having never been tested for HIV, 49.5% reported no worry about getting HIV, and 72.8% reported no worry that they may have already been exposed to HIV. The majority of participants (62.6%) reported inconsistent condom use and 25.2% reported sex with multiple partners. Correlations, means, and standard deviations for measures of interest are reported in Table 3.

**Table 3:** Correlations and descriptive statistics for key study variables

Variables	1	2	3	4	5	6	7	8	9	10
1. Age	–									
2. DUDIT	.071	–								
3. AUDIT	.030	.212**	–							
4. Medical Mistrust	− .037	− .024	.084	–						
5. Self-Efficacy	.133*	− .256**	− .051	.229**	–					
6. HIV Knowledge	.320**	.037	− .035	.004	.163*	–				
7. PrEP Knowledge	.201**	.084	− .089	− .101	.066	.362**	–			
8. Everyday Discrimination	− .125*	.431**	.223**	.059	− .257**	− .157**	.063	–		
9. PrEP Stereotypes	.078	.047	.019	.179**	− .046	− .070	− .141*	.120*	–	
10. PrEP Disapproval	− .112	− .037	− .025	− .005	− .203**	− .008	− .154*	.142*	.152*	
Means	34.04	19.02	4.74	24.17	36.67	6.95	2.64	30.16	12.01	6.57
SD	9.37	12.26	3.19	5.21	7.91	2.21	1.96	11.78	3.19	2.08

### Simple Regressions

#### Interest in Using Daily Oral PrEP

Regression results are shown in Table 4. Controlling for PrEP awareness, the following measures were associated with interest in daily oral PrEP (*p* < 0.05): lower scores on PrEP User Stereotypes [*b* = − 0.06 (SE = 0.02), *p* = 0.009] and PrEP Disapproval by Others [*b* = − 0.20 (SE = 0.03), *p* < 0.001], and higher PrEP Knowledge scores [*b* = 0.10 (SE = 0.04), *p* = 0.024]. Everyday Discrimination Scale scores predicted interest in PrEP only when controlling additionally for drug use severity and worry about HIV [*b* = − 0.01 (SE = 0.01), *p* = 0.040].

**Table 4:** Simple regression predicting interest in daily oral PrEP

	(1)	(2)	(3)	(4)	(5)	(6)	(7)	(8)	(9)
(Intercept)	3.79 (0.29) ***	4.48 (0.24)***	2.90 (0.21)***	2.40 (0.20)***	3.09 (0.36)***	3.47 (0.24)***	2.92 (0.12)***	2.88 (0.29)***	2.96 (0.35)***
PrEP user									
stereotypes	− 0.06 (0.02)**								
PrEP disapproval									
by others		− 0.20 (0.03)***							
Everyday Discrimination			0.01 (0.01)	− 0.01 (0.01)*					
Medical mistrust					0.00 (0.01)				
HIV Knowledge						− 0.06 (0.03)			
PrEP Knowledge							0.10 (0.04)*		
Health literacy								0.02 (0.02)	
Self-efficacy									0.00 (0.01)
Awareness of PrEP	0.09 (0.14)	− 0.04 (0.14)	0.08 (0.14)	− 0.08 (0.14)	0.09 (0.15)	0.14 (0.15)	− 0.12 (0.17)	0.08 (0.15)	0.09 (0.15)
Drug Use Severity				0.03 (0.01)***					
Worry about HIV				0.32 (0.06)***					
N	270	270	270	259	270	270	270	270	270
R^2^	0.027	0.127	0.005	0.184	0.002	0.014	0.021	0.004	0.002
F	3.653	19.403	0.713	14.304	0.205	1.828	2.798	0.470	0.272
RMSE	1.17	1.10	1.18	1.06	1.18	1.17	1.17	1.18	1.18

Estimate (standard error)

^*^p < 0.05, **p < 0.01, ***p < 0.001

#### Willingness to Take LAI PrEP

Controlling for LAI PrEP awareness, PrEP Disapproval by Others was the only measure associated with willingness to take LAI [*b* =  − 0.12 (SE = 0.03), *p* < 0.001] (Table 5).

**Table 5:** Simple regression predicting willingness to take long acting injectable

	(1)	(2)	(3)	(4)	(5)	(6)	(7)	(8)
(Intercept)	3.37 (0.26)***	3.89 (0.21)***	3.07 (0.18)***	3.06 (0.31)***	3.09 (0.22)***	2.99 (0.11)***	3.07 (0.26)***	3.14 (0.32)***
PrEP user stereotypes	− 0.02 (0.02)							
PrEP disapproval by others		− 0.12 (0.03)***						
Everyday Discrimination			0.00 (0.01)					
Medical mistrust				0.00 (0.01)				
HIV Knowledge					0.00 (0.03)			
PrEP Knowledge						0.04 (0.03)		
Health literacy							0.00 (0.02)	
Self-efficacy								0.00 (0.01)
Awareness of LAI	0.38 (0.16)*	0.38 (0.15)*	0.39 (0.16)*	0.40 (0.16)*	0.40 (0.16)*	0.36 (0.16)*	0.40 (0.16)*	0.39 (0.16)*
N	270	270	270	270	270	270	270	270
R^2^	0.028	0.079	0.023	0.023	0.023	0.028	0.023	0.023
F	3.857	11.398	3.184	3.180	3.178	3.905	3.179	3.191
RMSE	1.06	1.03	1.06	1.06	1.06	1.06	1.06	1.06

Estimate (standard error)

^*^p < 0.05, **p < 0.01, ***p < 0.001

### Multiple Regressions

#### Interest in Using Daily Oral PrEP

Tier one analyses retained the following predictors of interest in PrEP, controlling for PrEP awareness: PrEP User Stereotypes and PrEP Disapproval by Others (additionally controlling for insurance status, drug use severity, worry about HIV, and time since last tested for HIV), PrEP Knowledge (controlling for insurance status and worry about HIV), and Sexual Self-Efficacy (controlling for drug use severity). These regression estimates are shown in Table S2 in Supplementary Materials.

When these winning variables were entered in the Tier two analyses, again controlling for PrEP awareness, PrEP Disapproval by Others was significantly related to interest in PrEP [*b* = − 0.20 (SE = 0.03), *p* < 0.001; Table S6 in Supplementary Materials]. However, PrEP User Stereotypes [*b* = − 0.06 (SE = 0.02), *p* = 0.003], PrEP Disapproval by Others [*b* = − 0.15 (SE = 0.03), *p* < 0.001], and PrEP Knowledge [*b* = 0.10 (SE = 0.04), *p* = 0.013] became significantly associated with interest in PrEP when all relevant confounders were additionally accounted for (Table 6).

**Table 6:** Multiple regression predicting interest in daily oral PrEP: Tier two

	Adjusted
(Intercept)	3.55 (0.51)***
PrEP user stereotypes	− 0.06 (0.02)**
PrEP disapproval by others	− 0.15 (0.03)***
Knowledge of PrEP	0.10 (0.04)*
Self-efficacy	0.00 (0.01)
Awareness of PrEP	− 0.39 (0.15)*
Drug Use Severity	0.02 (0.01)***
Worry about HIV	0.29 (0.05)***
N	259
*R*^2^	0.311
F	
RMSE	0.98

Estimate (standard error)

^*^p < 0.05, **p < 0.01, ***p < 0.001

#### Willingness to Take Long Acting Injectable PrEP

Controlling for LAI PrEP awareness, tier one analyses retained these predictors (Table S3 in Supplementary Materials): PrEP Disapproval by Others (additionally controlling for insurance status and drug use severity), PrEP Knowledge (controlling for insurance status). Sexual Self-Efficacy was not retained by the models; however, it was still entered in tier two as specified in the methods.

Out of these variables, only PrEP Disapproval by Others was significantly related. This was true in both unadjusted [*b* = − 0.13 (SE = 0.03), *p* < 0.001; Table S7 in Supplementary Materials] and adjusted models [*b* = − 0.12 (SE = 0.03), *p* < 0.001; Table 7].

**Table 7:** Multiple regression predicting willingness to take long acting injectable: Tier two

	Adjusted
(Intercept)	3.74 (0.46)***
PrEP disapproval by others	− 0.12 (0.03)***
Knowledge of PrEP	0.02 (0.03)
Self-efficacy	0.00 (0.01)
Awareness of LAI	0.31 (0.16)*
Drug Use Severity	0.01 (0.01)*
N	270
*R*^2^	0.103
F	6.070
RMSE	1.02

Estimate (standard error)

^*^p < 0.05, **p < 0.01, ***p < 0.001

## Discussion

The current study explored predisposing, enabling, and reinforcing factors as predictors of interest in using daily oral PrEP and willingness to use LAI PrEP. Our hypothesis that these factors would emerge as significant predictors of interest in and willingness to use PrEP was partially supported.

In examining predisposing factors, we included measures of HIV knowledge, PrEP knowledge, and health literacy. Among these measures, in the simple regression models, only PrEP knowledge emerged as a predictor of interest in using daily oral PrEP while HIV knowledge and health literacy did not predict interest. The Information-Motivation-Behavioral Skills (IMB) model is a widely-cited model for understanding behavior change and is frequently used as a framework for HIV prevention intervention development (Fisher et al., 2009). These findings are consistent with the IMB model of health behavior change which posits that information (knowledge) related to the behavior targeted for change is an essential first element (Ni et al., 2021). However, in the multiple regression model, the significant association did not hold when other variables were included in the model. Prior research has indicated that information may not directly affect behavior change when the health behavior requires multiple skills to accomplish it, such as with PrEP initiation (McFarland et al., 2020). Consistent with this, findings in the current study support that multiple variables impact interest in using PrEP.

Sexual self-efficacy which was included as a potential enabling factor was not found to be a predictor of interest in using daily oral PrEP or willingness to use LAI PrEP in any of the models. This is contrary to an earlier study that found that improvements in self-efficacy over time predicted reductions in the behaviors that make PWUD more susceptible to HIV (Kang et al., 2004). Subsequent research on the importance of self-efficacy in the HIV care continuum has found that, for PWUD, domain-specific self-efficacy components should be measured to accurately predict the impact of self-efficacy on HIV prevention and treatment behaviors with researchers calling for multi-domain assessments of self-efficacy (Starks et al., 2022). As a single-domain measure of sexual behavior self-efficacy was used for the current study, it is possible that other aspects of self-efficacy which were not measured may be more important in predicting PrEP interest and willingness.

Stigma (a potential reinforcing factor) emerged as a consistent predictor with higher scores in PrEP user stereotypes associated with lesser interest in daily oral PrEP in both the simple regression model and the multiple regression model. Similarly, higher anticipated disapproval from others about PrEP use predicted lesser interest in using daily oral PrEP and willingness to take LAI PrEP in both the simple and multiple regression models.

In previously published studies, researchers have noted that stigma can impact both the degree to which HIV prevention efforts are used and the effectiveness of such efforts. Stigma is defined as an attribute held by a person that results in a negative judgment toward that person (Goffman, 2009). This is exemplified in the current study as individuals who hold stereotypes regarding people who use PrEP were less likely to be interested in PrEP themselves. Similarly, anticipating disapproval from family, friends, or partners predicted less interest and willingness to use daily oral PrEP and LAI PrEP. This is consistent with previous findings that anticipation of disapproval by others negatively impacts HIV testing and PrEP use (Heads et al., 2021).

Anticipation of PrEP stigma is a potential barrier to PrEP initiation among members of marginalized populations. People who use PrEP are often stereotyped, resulting in discouragement of engagement in the PrEP care continuum at multiple levels (interest, uptake, initiation, adherence) (Calabrese, 2020). It is worth noting that, although several studies have investigated factors associated with interest in and willingness to use PrEP, these studies have primarily focused on men who have sex with men. The current study broadens the knowledge related to interest in and willingness to use PrEP by including a diverse sample of individuals with problematic substance use and specifically focusing on both daily oral and LAI PrEP formulations.

PrEP awareness remains low overall. Approximately half of the current sample reported that they had never heard of PrEP prior to the survey. This is somewhat higher than a previous study of PrEP awareness among people in treatment for a substance use disorder (Ni et al., 2021) and consistent with previous studies reporting a similar level of awareness of PrEP among people who use drugs who were not in treatment (Heads et al., 2021; McFarland et al., 2020). This may be evidence of the potential role of addiction treatment facilities in educating and increasing familiarity with the benefits and availability of PrEP.

### Implications for Intervention and Future Research

The results have implications for the development of future interventions. Although about half of the participants were aware of PrEP, high levels of anticipated PrEP stigma and disapproval by others were identified. Stigmatizing attitudes toward others and anticipated stigma from friends and partners would likely interfere with willingness to begin the first steps toward using PrEP as an HIV prevention tool.

Our findings highlight two important areas for improving engagement in the PrEP care cascade. First, to effectively end the HIV epidemic and engage all who can benefit from PrEP, it is important to address and reduce stigma associated with HIV and PrEP. Second, given that anticipated disapproval from others was a significant predictor of interest in PrEP and willingness to use LAI PrEP, social support may also be an important factor to address as positive support from trusted relationships can reduce the impact of stigma on decisions to use PrEP (Brooks et al., 2019).

Previous researchers have suggested stigma-reducing interventions as an important step toward increasing PrEP initiation (Kelley et al., 2015). PWUD may benefit from HIV prevention interventions that specifically address perceptions of stigma associated with PrEP use. Researchers have called for large-scale interventions that systematically address stigma (Nyblade et al., 2021). Specifically, interventions should be designed to increase awareness of stigma and confront stigmatizing attitudes. Perhaps, most importantly, those individuals who are most likely to experience stigma should be included in the plan for implementing stigma-reducing programs.

The findings support the importance of designing behavioral interventions to address PrEP stigma among individuals with problematic substance use. Existing behavioral interventions that are effective for reducing behaviors that increase susceptibility for HIV include education on personal risk for HIV, practical skills training for condom negotiation, refusal skills, and communicating with partners. These interventions do not include strategies for managing the effects of stigma.

New PrEP formulations, such as LAI, offer alternatives to the daily oral pill and may offer more concealable options, thus alleviating concerns about disapproval from others (Kerrigan et al., 2018; Murray et al., 2018; Philbin et al., 2021). Targeted media campaigns can be important tools to decrease PrEP stigma due to its potential to increase awareness while normalizing HIV prevention and PrEP use.

### Limitations

The current study’s cross-sectional design does not allow assessment of causality. Our sample included adults living in Harris County, TX with self-reported problematic alcohol or illicit substance use or a diagnosed substance use disorder. Due to the reliance on self-report instruments, it is not possible to verify substance use or substance use severity. We limited the study to the specific geographic area, to capture responses from residents of this EHE priority jurisdiction. Therefore, the results are not generalizable to other regions of the US. A further limitation is that our study focused on PrEP user stereotypes and PrEP disapproval by others using a single instrument. Stigma related to substance use was not explored in this study and likely also contributes to hesitancy to engage in PrEP care. Future research should include a measure of substance use stigma and qualitatively explore PrEP stigma with this population to determine what aspects of stigma are most salient for this population. As mentioned earlier, the use of a single-domain instrument of sexual behavior self-efficacy is a limitation of the current study. Future studies should consider using a multidimensional measure of self-efficacy which includes a measure specific to PrEP self-efficacy. Additionally, the current study focused solely on predictors of interest in and willingness to use PrEP and not actual PrEP uptake and initiation.

### Summary and Conclusions

PrEP is an important biomedical HIV prevention tool in the EHE plan for the US. However, PrEP uptake has been slow and still does not reach all who could benefit from it. PrEP stigma is a barrier to PrEP uptake, initiation, and adherence. A far-reaching and systemic approach to addressing stigma is needed to remove individual, interpersonal, and structural barriers to PrEP care among people with problematic substance use.

## Supplementary Information

Below is the link to the electronic supplementary material.

Supplementary file1 (DOCX 40 KB)

## Data Availability

Data are available from the corresponding author upon reasonable request.
