# Supplementary material for: Interest in and Willingness to Use PrEP: A Cross-Sectional Study of Individuals with Problematic Substance Use Residing in a High HIV Prevalence Jurisdiction
Source: Arch Sex Behav. 2024 Jul 17;53(9):3687–98. doi: 10.1007/s10508-024-02936-z (PMC11390792; doi:10.1007/s10508-024-02936-z)
Supplement: Supplementary file 1 — Supplementary file1 (DOCX 40 KB) [file 10508_2024_2936_MOESM1_ESM.docx]

Interest and Willingness to Use PrEP: A Cross Sectional Study of Individuals with Problematic Substance use Residing in a High HIV Prevalence Jurisdiction

Supplementary Materials

## Supplementary Results

***Table S1. Confounder testing table***

|  | | | | | | | | | | | Covary characteristic when predicting y in multiple regression?^ | | | | | |
| --- | --- | --- | --- | --- | --- | --- | --- | --- | --- | --- | --- | --- | --- | --- | --- | --- |
| **Characteristic, p-value** | **x (predictor)** | | | | | | | | **y (outcome)** | | Discrimination/Stigma: prepster, predis, edsscore, MMItot | | Medical knowledge: Hktot, Pkscore, healthlit | | Self-efficacy: SETot | |
|  | **prepster** | **predis** | **edsscore** | **SEtot** | **MMItot** | **HKtot** | **PKscore** | **healthlit** | **will1** | **will_lai** | will1 | will_lai | will1 | will_lai | will1 | will_lai |
| Age | 0.201 | 0.065 | 0.040 | 0.029 | 0.543 | <.001 | <.001 | 0.511 | 0.220 | 0.938 |  |  |  |  |  |  |
| Sex | 0.900 | 0.572 | 0.636 | 0.749 | 0.223 | 0.452 | 0.523 | 0.491 | 0.010 | 0.347 |  |  |  |  |  |  |
| Sexual Orientation* | 0.268 | 0.221 | 0.419 | 0.657 | 0.010 | 0.006 | <.001 | 0.485 | 0.117 | 0.304 |  |  |  |  |  |  |
| Hispanic Ethnicity | 0.098 | 0.580 | 0.669 | 0.073 | 0.276 | 0.173 | 0.027 | 0.092 | 0.127 | 0.288 |  |  |  |  |  |  |
| Race* | 0.534 | 0.332 | 0.815 | 0.786 | 0.023 | 0.851 | 0.327 | 0.167 | 0.057 | 0.627 |  |  |  |  |  |  |
| Education | 0.611 | 0.166 | 0.875 | 0.157 | 0.061 | 0.441 | 0.784 | 0.008 | 0.209 | 0.345 |  |  |  |  |  |  |
| Insurance* | 0.184 | 0.573 | 0.016 | 0.160 | 0.008 | 0.066 | 0.488 | <.001 | <.001 | 0.004 | yes | yes | yes | yes |  |  |
| DUDIT | 0.440 | 0.541 | <.001 | <.001 | 0.699 | 0.544 | 0.167 | 0.673 | <.001 | 0.004 | yes | yes |  |  | yes | yes |
| Worry about HIV | 0.133 | 0.027 | <.001 | 0.076 | 0.389 | 0.006 | 0.969 | 0.581 | <.001 | 0.054 | yes |  | yes |  |  |  |
| Condom Use | 0.735 | 0.056 | 0.008 | 0.904 | 0.404 | 0.002 | 0.566 | 0.472 | 0.493 | 0.518 |  |  |  |  |  |  |
| Aware of PrEP | 0.882 | 0.011 | 0.233 | 0.213 | 0.076 | 0.006 | <.001 | 0.121 | 0.522 | 0.570 | yes |  | yes |  | yes |  |
| Aware of LAI | 0.213 | 0.604 | 0.007 | 0.007 | 0.079 | 0.092 | 0.002 | 0.041 | 0.082 | 0.012 |  | yes |  | yes |  | yes |
| HIV Ever Tested | 0.981 | 0.953 | 0.888 | <.001 | 0.661 | 0.008 | 0.003 | 0.131 | 0.789 | 0.721 |  |  |  |  |  |  |
| HIV Last Tested* | 0.123 | 0.617 | 0.053 | 0.151 | 0.005 | 0.131 | 0.941 | 0.074 | 0.017 | 0.187 | yes |  |  |  |  |  |

Item legend:

prepster = PrEP user sterotypes

predis = PrEP disapproval

Edsscore = Experiences of discrimination

SEtot = self-efficacy

MMItot = Medical mistrust

HKtot = Knowledge of HIV risk

PKscore = Knowledge of PrEP

Healthlit = health literacy

will1 = interested in PrEP

will_lai = Willing to take LAI

^A characteristic was considered a confounder if it is significantly related to BOTH the outcome AND AT LEAST ONE of the predictors.

All p-values derived from models used lm() or glm() in R. *p-values were taken from chi-squared test when categorical predictor had >2 levels

^For simple regression models focused on an individual predictor, include characteristic as covariate if it sig. related to both outcome and that predictor

In addition, covary Aware of PrEP when predicting will1 or will2; similarly, covary Aware of LAI when predicting will_lai or will_lai2.

e.g.,

will1 ~ PrEP_Aware + HIVworry + predis

will2 ~ PrEP_Aware + race + MMItot

Table S2: Multiple Regression Predicting Interest in Daily Oral PrEP - Tier one

|  | Reinforcing (Discrimination/ Stigma) | | Predisposing (Medical knowledge) | | Enabling (Self-efficacy) | |
| --- | --- | --- | --- | --- | --- | --- |
|  | Unadjusted | Adjusted | Unadjusted | Adjusted | Unadjusted | Adjusted |
| (Intercept) | 4.63 (0.35)*** | 4.47 (0.45)*** | 3.47 (0.24)*** | 1.90 (0.19)*** | 3.08 (0.10)*** | 2.06 (0.39)*** |
| PrEP user stereotypes | -0.04 (0.02)* | -0.09 (0.03)*** |  |  |  |  |
| PreP disapproval by others | -0.20 (0.03)*** | -0.15 (0.04)*** |  |  |  |  |
| Experiences  of discrimination | 0.01 (0.01)* |  |  |  |  |  |
| Knowledge of HIV risk |  |  | -0.09 (0.03)** |  |  |  |
| Knowledge of PrEP |  |  | 0.14 (0.05)** | 0.14 (0.04)*** |  |  |
| Self-efficacy |  |  |  |  |  | 0.01 (0.01) |
| Awareness of PrEP | -0.06 (0.14) | -0.05 (0.16) | -0.14 (0.17) | -0.29 (0.16) | 0.09 (0.14) | -0.02 (0.14) |
| Insurance: Medicaid/ Medicare/Harris Health |  | 0.32 (0.21) |  | 0.47 (0.18)* |  |  |
| Insurance: Private |  | 0.40 (0.20)* |  | 0.45 (0.17)** |  |  |
| Drug Use Severity |  | 0.02 (0.01)* |  |  |  | 0.03 (0.01)*** |
| Worry about HIV |  | 0.27 (0.06)*** |  | 0.32 (0.05)*** |  |  |
| HIV tested: 6 months to  less than 1 year ago |  | -0.46 (0.22)* |  |  |  |  |
| HIV tested: More than year ago |  | -0.53 (0.19)** |  |  |  |  |
| N | 270 | 160 | 270 | 252 | 270 | 270 |
| R2 | 0.155 | 0.379 | 0.047 | 0.188 | 0.002 | 0.077 |
| F | 12.130 |  | 4.400 |  | 0.411 | 7.356 |
| RMSE | 1.09 | 0.93 | 1.15 | 1.06 | 1.18 | 1.14 |
| Estimate (standard error). | | | | | | |
| * p < 0.05, ** p < 0.01, *** p < 0.001 | | | | | | |

Table S3: Multiple Regression Predicting Willingness to take LAI - Tier one

|  | Reinforcing (Discrimination/ Stigma) | | Predisposing (Medical knowledge) | | Enabling (Self-efficacy) | |
| --- | --- | --- | --- | --- | --- | --- |
|  | Unadjusted | Adjusted | Unadjusted | Adjusted | Unadjusted | Adjusted |
| (Intercept) | 3.89 (0.21)*** | 3.54 (0.25)*** | 3.08 (0.07)*** | 2.75 (0.15)*** | 3.08 (0.07)*** | 2.83 (0.12)*** |
| PreP disapproval by others | -0.12 (0.03)*** | -0.13 (0.03)*** |  |  |  |  |
| Knowledge of PrEP |  |  |  | 0.05 (0.03) |  |  |
| Awareness of LAI | 0.38 (0.15)* | 0.26 (0.15) | 0.40 (0.16)* | 0.27 (0.16) | 0.40 (0.16)* | 0.36 (0.16)* |
| Insurance: Medicaid/ Medicare/Harris Health |  | 0.43 (0.17)** |  | 0.49 (0.17)** |  |  |
| Insurance: Private |  | 0.26 (0.15) |  | 0.27 (0.16) |  |  |
| Drug Use Severity |  | 0.01 (0.01) |  |  |  | 0.01 (0.01)** |
| N | 270 | 263 | 270 | 263 | 270 | 270 |
| R2 | 0.079 | 0.127 | 0.023 | 0.056 | 0.023 | 0.049 |
| F | 11.398 |  | 6.377 |  | 6.377 | 6.842 |
| RMSE | 1.03 | 0.98 | 1.06 | 1.02 | 1.06 | 1.05 |
| Estimate (standard error). | | | | | | |
| * p < 0.05, ** p < 0.01, *** p < 0.001 | | | | | | |

Table S4: Multiple Regression Predicting Interest in Daily Oral PrEP - Full models

|  | Reinforcing (Discrimination/ Stigma) | | Predisposing (Medical knowledge) | | Enabling (Self-efficacy) | |
| --- | --- | --- | --- | --- | --- | --- |
|  | Unadjusted | Adjusted | Unadjusted | Adjusted | Unadjusted | Adjusted |
| (Intercept) | 4.62 (0.45)*** | 4.42 (0.60)*** | 3.26 (0.36)*** | 2.11 (0.37)*** | 2.96 (0.35)*** | 2.06 (0.39)*** |
| PrEP user stereotypes | -0.04 (0.02)* | -0.09 (0.03)*** |  |  |  |  |
| PreP disapproval by others | -0.20 (0.03)*** | -0.15 (0.04)*** |  |  |  |  |
| Experiences  of discrimination | 0.01 (0.01)* | 0.00 (0.01) |  |  |  |  |
| Medical mistrust | 0.00 (0.01) | 0.00 (0.02) |  |  |  |  |
| Knowledge of HIV risk |  |  | -0.09 (0.03)** | -0.03 (0.03) |  |  |
| Knowledge of PrEP |  |  | 0.14 (0.05)** | 0.15 (0.04)*** |  |  |
| Health literacy |  |  | 0.02 (0.02) | 0.00 (0.02) |  |  |
| Self-efficacy |  |  |  |  | 0.00 (0.01) | 0.01 (0.01) |
| Awareness of PrEP | -0.06 (0.14) | -0.05 (0.16) | -0.15 (0.17) | -0.29 (0.16) | 0.09 (0.15) | -0.02 (0.14) |
| Insurance: Medicaid/ Medicare/Harris Health |  | 0.32 (0.21) |  | 0.43 (0.19)* |  |  |
| Insurance: Private |  | 0.40 (0.20) |  | 0.43 (0.17)* |  |  |
| Drug Use Severity |  | 0.02 (0.01)* |  |  |  | 0.03 (0.01)*** |
| Worry about HIV |  | 0.26 (0.07)*** |  | 0.31 (0.06)*** |  |  |
| HIV tested: 6 months to  less than 1 year ago |  | -0.47 (0.23)* |  |  |  |  |
| HIV tested: More than year ago |  | -0.53 (0.19)** |  |  |  |  |
| N | 270 | 160 | 270 | 252 | 270 | 270 |
| R2 | 0.155 | 0.380 | 0.050 | 0.191 | 0.002 | 0.077 |
| F | 9.668 |  | 3.452 |  | 0.272 | 7.356 |
| RMSE | 1.09 | 0.93 | 1.15 | 1.05 | 1.18 | 1.14 |
| Estimate (standard error). | | | | | | |
| * p < 0.05, ** p < 0.01, *** p < 0.001 | | | | | | |

Table S5: Multiple Regression Predicting Willingness to take LAI - Full models

|  | Reinforcing (Discrimination/ Stigma) | | Predisposing (Medical knowledge) | | Enabling (Self-efficacy) | |
| --- | --- | --- | --- | --- | --- | --- |
|  | Unadjusted | Adjusted | Unadjusted | Adjusted | Unadjusted | Adjusted |
| (Intercept) | 3.90 (0.41)*** | 3.65 (0.43)*** | 3.08 (0.33)*** | 2.81 (0.33)*** | 3.14 (0.32)*** | 2.66 (0.36)*** |
| PrEP user stereotypes | -0.01 (0.02) | -0.02 (0.02) |  |  |  |  |
| PreP disapproval by others | -0.12 (0.03)*** | -0.12 (0.03)*** |  |  |  |  |
| Experiences  of discrimination | 0.00 (0.01) | 0.00 (0.01) |  |  |  |  |
| Medical mistrust | 0.00 (0.01) | 0.01 (0.01) |  |  |  |  |
| Knowledge of HIV risk |  |  | -0.02 (0.03) | -0.01 (0.03) |  |  |
| Knowledge of PrEP |  |  | 0.05 (0.04) | 0.05 (0.04) |  |  |
| Health literacy |  |  | 0.00 (0.02) | 0.00 (0.02) |  |  |
| Self-efficacy |  |  |  |  | 0.00 (0.01) | 0.00 (0.01) |
| Awareness of LAI | 0.35 (0.16)* | 0.27 (0.15) | 0.34 (0.16)* | 0.26 (0.16) | 0.39 (0.16)* | 0.37 (0.16)* |
| Insurance: Medicaid/ Medicare/Harris Health |  | 0.46 (0.17)** |  | 0.49 (0.18)** |  |  |
| Insurance: Private |  | 0.29 (0.16) |  | 0.28 (0.16) |  |  |
| Drug Use Severity |  | 0.01 (0.01)* |  |  |  | 0.01 (0.01)** |
| N | 270 | 263 | 270 | 263 | 270 | 270 |
| R2 | 0.082 | 0.135 | 0.030 | 0.056 | 0.023 | 0.050 |
| F | 4.724 |  | 2.018 |  | 3.191 | 4.630 |
| RMSE | 1.03 | 0.98 | 1.06 | 1.02 | 1.06 | 1.04 |
| Estimate (standard error). | | | | | | |
| * p < 0.05, ** p < 0.01, *** p < 0.001 | | | | | | |

Table S6:Multiple Regression Predicting Interest in Daily Oral PrEP - Tier two

|  | Winning variables | |
| --- | --- | --- |
|  | Unadjusted | Adjusted |
| (Intercept) | 5.01 (0.50)*** | 3.55 (0.51)*** |
| PrEP user stereotypes | -0.03 (0.02) | -0.06 (0.02)** |
| PrEP disapproval by others | -0.20 (0.03)*** | -0.15 (0.03)*** |
| Knowledge of PrEP | 0.07 (0.04) | 0.10 (0.04)* |
| Self-efficacy | -0.01 (0.01) | 0.00 (0.01) |
| Awareness of PrEP | -0.17 (0.16) | -0.39 (0.15)* |
| Drug Use Severity |  | 0.02 (0.01)*** |
| Worry about HIV |  | 0.29 (0.05)*** |
| N | 270 | 259 |
| R^2^ | 0.149 | 0.311 |
| F | 9.262 |  |
| RMSE | 1.09 | 0.98 |
| Estimate (standard error). | | |
| * p < 0.05, ** p < 0.01, *** p < 0.001 | | |

Table S7: Multiple Regression Predicting Willingness to take LAI - Tier two

|  | Winning variables | |
| --- | --- | --- |
|  | Unadjusted | Adjusted |
| (Intercept) | 4.19 (0.42)*** | 3.74 (0.46)*** |
| PrEP disapproval by others | -0.13 (0.03)*** | -0.12 (0.03)*** |
| Knowledge of PrEP | 0.02 (0.03) | 0.02 (0.03) |
| Self-efficacy | -0.01 (0.01) | 0.00 (0.01) |
| Awareness of LAI | 0.33 (0.16)* | 0.31 (0.16)* |
| Drug Use Severity |  | 0.01 (0.01)* |
| N | 270 | 270 |
| R^2^ | 0.084 | 0.103 |
| F | 6.087 | 6.070 |
| RMSE | 1.03 | 1.02 |
| Estimate (standard error). | | |
| * p < 0.05, ** p < 0.01, *** p < 0.001 | | |
